# Single session of pattern scanning laser versus multiple sessions of conventional laser for panretinal photocoagulation in diabetic retinopathy: Efficacy, safety and painfulness

**DOI:** 10.1371/journal.pone.0219282

**Published:** 2019-07-16

**Authors:** Jan Nemcansky, Alexandr Stepanov, Sabina Nemcanska, Petr Masek, Hana Langrova, Jan Studnicka

**Affiliations:** 1 Faculty Hospital Ostrava, Department of Ophthalmology, Ostrava, Czech Republic; 2 University of Ostrava, Faculty of Medicine, Department of Craniofacial Sciences, Ostrava, Czech Republic; 3 Faculty Hospital Hradec Kralove, Department of Ophthalmology, Hradec Kralove, Czech Republic; 4 Charles University, Faculty of Medicine in Hradec Kralove, Department of Ophthalmology, Hradec Kralove, Czech Republic; University of Debrecen, Faculty of Medicine, HUNGARY

## Abstract

**Purpose:**

To evaluate the clinical efficiency, safety and painfulness of retinal laser photocoagulation employing a pattern scanning laser system Pascal given in a single-session versus conventional laser multiple-session treatment of the same patient with diabetic retinopathy during 12-month follow-up.

**Methods:**

The cohort included 60 eyes in 30 patients treated at the Ophthalmology Clinic, Faculty Hospital Ostrava, from 2008 to 2013. Panretinal laser coagulation was performed on one eye using the multispot panretinal photocoagulation given in a single-session system Pascal (OptiMedica, Santa Clara, California). On the other eye laser treatment was carried out by the classic conventional multiple-session method.

**Results:**

The performance of Pascal panretinal laser coagulation was evaluated as significantly less painful (visual scale of pain was 3.28 ± 1.9) than the performance of conventional photocoagulation (visual scale of pain was 3.93 ± 1.88) with similar efficiency. Distribution of progression of diabetic retinopathy in individual patients was very similar in both groups under comparison, and was strictly paired in 24 of the 30 patients at the end of 1-year follow-up.

**Conclusion:**

Laser photocoagulation of the retina with the use of short impulse durations and patterns in patients with diabetic retinopathy given in one session possesses similar efficiency to that of conventional retinal photocoagulation in multiple sessions. The single session treatment is also better tolerated by patients and in addition to this, it shortens the performance of the whole therapy, which potentially saves considerable funds of all subjects participating in the process of treatment.

**Trial registration:**

ClinicalTrials.gov NCT03672656.

## Introduction

Photocoagulation of the retina by laser has been one of the traditional therapeutic techniques employed in ophthalmology since the mid-20^th^ century. The actual mechanism of the effect of laser in photocoagulation is absorption of light energy, its conversion into heat in the retinal epithelium pigment (RPE) and in the outer layers of the retina, thermal destruction of some RPE in the ischemic retina, and a resultant increase in oxygen tension benefitting the remaining tissue. The most frequent application of laser photocoagulation of the retina both in the past and today has been the treatment of vascular diseases of the retina–diabetic retinopathies.

The protocol from both the Diabetic Retinopathy Study (DRS) and the Early Treatment Diabetic Retinopathy Study (ETDRS) has been considered the standard technique for the performance of laser photocoagulation of the retina till now. This protocol was based on the technical preconditions of available laser systems, and its efficiency was supported by therapeutic results and particularly the afore-mentioned randomized studies [1, 2]. In the past decade new technologies have been introduced into industry and medicine, resulting in the development of a new generation of laser systems. These are able to produce individual laser impulses shorter by an order of magnitude (0.01 s and shorter). At the same time these impulses are directed in a quick sequence one after another in so-called patterns. A very important innovation is the possibility of producing the individual discharges in certain pre-adjusted patterns by means of a microprocessor-controlled scanner. The system is able to produce within a tenth of a second up to tens of impulses. Because of these properties the technique is described as semiautomatic. The first commercially available laser of this class was the so-called PASCAL (PAttern SCAnning Laser) [3]. These innovations make it possible to change standard therapeutic protocols because of laser system Pascal use is possible to shorten the performance of the whole therapy, and potentially to save considerable funds of all subjects participating in the process of treatment. However, no reliable data are available concerning either the short-term or long-term efficiency of treatments performed in this way, and particularly the safety, clinical efficiency and painfulness of these treatments.

This paper aims to evaluate the clinical efficiency, safety and painfulness of panretinal laser photocoagulation employing a laser system Pascal given in a single-session versus conventional laser multiple-session treatment of the same patient with diabetic retinopathy.

## Methods

In the period from 1 June 2008 to 30 June 2013, 60 eyes of 30 patients treated at the Ophthalmology Clinic, Faculty Hospital Ostrava, and both eyes of the same patient were treated. Inclusion criteria were patients with severe non-proliferative diabetic retinopathy (NPDR), or proliferative diabetic retinopathy (PDR). Exclusion criteria include previous retinal laser photocoagulation, vitrectomy or associated vascular retinal diseases. All patients signed informed consent form, and examination of the patients was approved by the Ethical Commission of the Faculty of Medicine of University in Ostrava and the University Hospital in Ostrava at 1 April 2008. The authors confirm that all ongoing and related trials for this study are registered in ClinicalTrials.gov. The registration was made with a delay due to lack of awareness.

**Fig 1:**
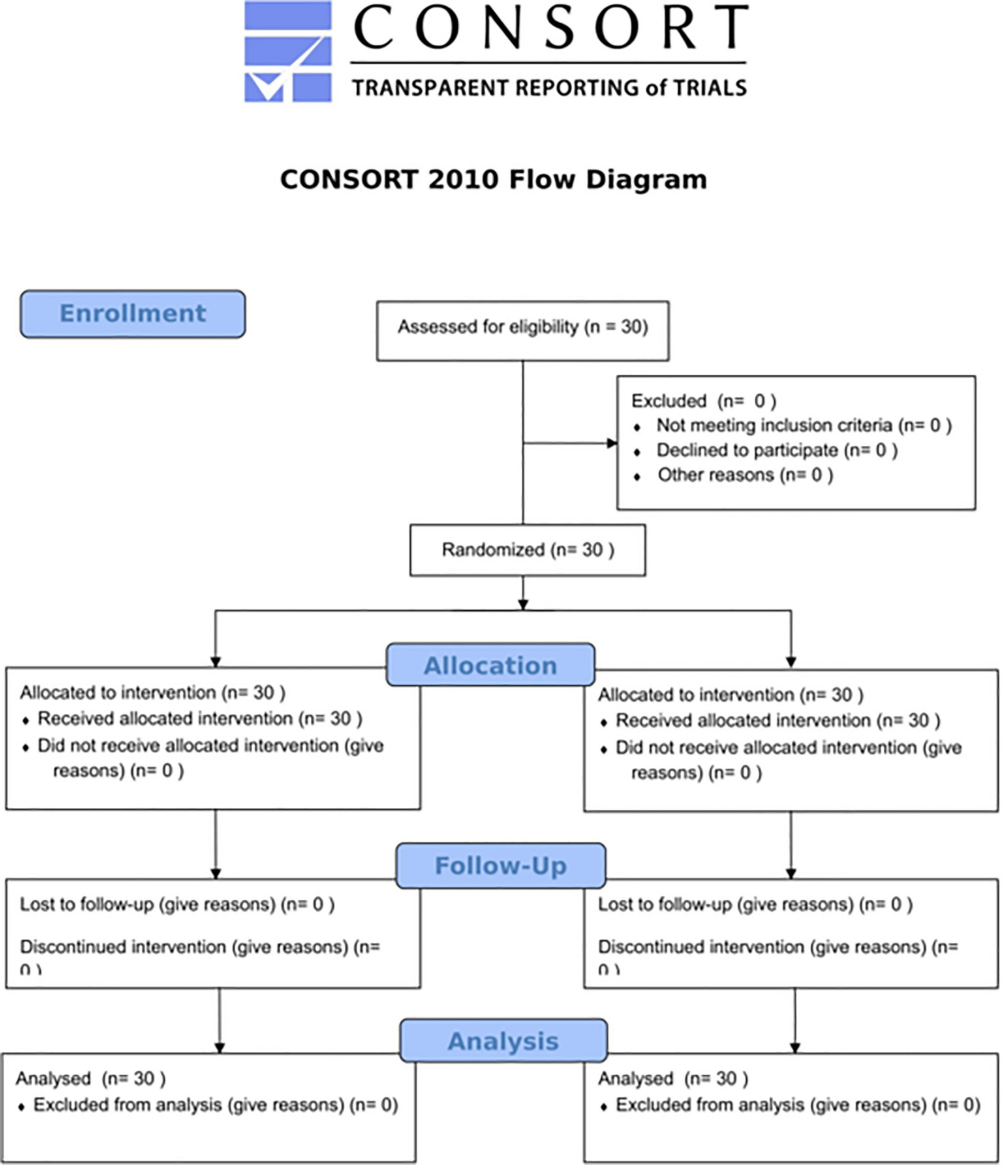
CONSORT flow diagram.

Thirty patients have met all of the inclusion criteria to be eligible for participation in this study and had a follow-up period of 12 months (Fig 1). In all patients, panretinal laser photocoagulation was delivered by Pascal Photocoagulator laser system (Topcon Medical Laser Systems, Inc., Santa Clara, CA, USA) at the same time on one eye in one or two episodes with the use of technical innovations (shortening of the time of the impulse, use of patterns), and on the other eye using the classic conventional method according to the standardized protocol ETDRS [1, 2, 4] (conventional laser coagulation). Allocation of the right or left eye to the individual type of selected photocoagulation of the retina was carried out using the biased coin method.

For all patients, case histories were recorded. Examination of the anterior segment was carried out with the use of a slit lamp. The posterior pole was examined biomicroscopically using a 90D Superfield NC lens (Volk Optical, Inc., Ohio, USA), and then a photograph was taken with a Carl Zeiss FF450 Plus IR camera with Visupac Digital Imaging System (Carl Zeiss Meditec, Inc., Dublin, California, USA); also using this camera fluorescent angiography was performed. At the same time spectral OCT of the macula was carried out using Spectralis OCT (Heidelberg Engineering, Inc., Carlsbad, California, USA). Additionally, a visual analogue scale of painfulness of 0–8, with 8 being the most painful was evaluated.

### Performance of Pascal panretinal laser photocoagulation

Laser photocoagulation of the retina was performed under local anaesthesia using a laser Pascal Photocoagulator. First, the laser energy necessary for the production of one laser spot was titrated: the length of an individual impulse was 20 ms, the spot size was 400 μm, and the laser energy was gradually increased until a grey laser spot was achieved. The pattern was then selected: in the treatment using panretinal photocoagulation (PRP) this was usually 3x3–5x5. This treatment was performed in one or two episodes/sessions.

### Performance of conventional laser photocoagulation

The first eye having been treated as above with the use of innovations, conventional treatment without use of innovations was then carried out on the patient’s second eye. Laser treatment of the retina was carried out according to the standardized protocol ETDRS, always under local anaesthesia and by means of a laser Pascal Photocoagulator. First, the laser energy necessary for the production of one laser spot was titrated: the length of an individual impulse was 0.2 s, the spot size was 400 μm, and the laser energy was gradually increased until a grey laser spot was achieved. If a grey colour could not be achieved by laser energy of 1000 mW, the impulse was prolonged by 0.1 s. The individual laser coagulations were then applied to achieve treatment of the area of the retina indicated for photocoagulation. This treatment was carried out in 4 individual episodes, always at one-week intervals.

We have followed the recommendations of the ICO guidelines for diabetic eye care 2018 (The International Council of Ophthalmology), where it is recommended to perform 1200–1600 burns in 1 to 3 sessions [5]. We perform of the average of 1685 burns (range 1400–2160) in this study, so the average number of sessions was higher. The reason for a higher number of laser burns was the predominance of the eyes with proliferative DR in the group of the patients.

The clinical effect was evaluated as the fulfilment of the purpose of laser photocoagulation of the retina–its stabilization. The same physician performed the laser treatment and graded the clinical effect over a 1-year follow-up period. Stabilization was evaluated as an improvement of the clinical finding in the fundus. The condition was evaluated as progression if there was deterioration of the clinical finding in the fundus (progression of neovascularisation or DME, hemophthalmus and traction retinal detachment). The subgroup “failure of treatment”, when despite another therapeutic intervention there was no stabilization of the disease and the condition could not be further influenced therapeutically, was excluded from this group. In case of progression of the disease, another therapeutic intervention (multiple repetition/supplementation of laser photocoagulation, pars plana vitrectomy, or anti-VEGF treatment) was carried out and the evaluation was terminated. In addition, the results of the evaluation of pain in the visual scale of pain were compared.

The methods of descriptive statistics were first employed to describe the variables. For continuous variables numerical characteristics (arithmetic mean, median, and standard deviation) were defined and a box graph was drawn. Nominal variables (sex, stabilization of the biomicroscopic finding, type of treatment, etc.) were described by absolute and relative frequencies. In addition, in continuous variables the Shapiro-Wilk normality test was carried out.

Nonparametric Kolmogorov-Smirnov test was performed at the level of significance of 0.05. Statistical analysis was performed using the software IBM SPSS Statistics version 22.

## Results

General characteristics of the cohort are presented in S1 Table. All patients completed 12-month follow-up. The distribution of diagnoses (NPDR, PDR) after the performance of a cross test is comparable in both groups (PASCAL, Conventional). Shapiro-Wilk normality test for the PASCAL group was 0.403, for the Conventional group 0.351, so data were normally distributed.

The laser energy employed in the individual eyes in treatment by the PASCAL method was 180–675 mW (mean 473 mW, median 486 mW, SD 128 mW), and the number of laser spots was 1600–2800 (mean 2113 spots, median 2089 spots, SD 328 spots). The laser energy used in the individual eyes in treatment by the Conventional method was 165–575 mW (mean 295 mW, median 285 mW, SD 96 mW), and the number of laser spots was 1400–2160 (mean 1685 spots, median 1683 spots, SD 179 spots). The number of laser spots and average energy were significantly higher in the PASCAL method than in the Conventional treatment.

### Efficiency of treatment

In the interval up to 6 months from the beginning of the follow-up period, stabilization of the clinical findings was observed in all patients included in the cohort. In the interval from 6 to 12 months from the beginning of the follow-up period, in the case of one patient both eyes showed progression of the disease, decreased vision and development of traction retinal detachment of the retina. S2 Table presents the efficiency of treatment at 12 months follow-up for both groups of patients. Distribution of progression during follow-up in individual patients was very similar in both groups and occurred in 18 eyes of 13 patients, 8 in the PASCAL group and 10 in the Conventional group.

Eyes with DME progression (2 eyes in Pascal group and 2 eyes in Conventional group) were treated by means of anti-VEGF drugs or by repeated laser photocoagulation. In eyes with progression of neovascularisation (2 eyes in Pascal group and 3 eyes in Conventional group) PRP was supplemented. Progression of the character of hemophthalmus occurred in 9 eyes of 6 patients, in 3 of whom both eyes were affected, and in the other 3, one eye was affected. This was 4 eyes treated by the PASCAL method and 5 eyes by the Conventional method. In these eyes PPV was performed, and in all patients the state was subsequently stabilized.

### Subjective evaluation of pain after laser coagulation

**Fig 2:**
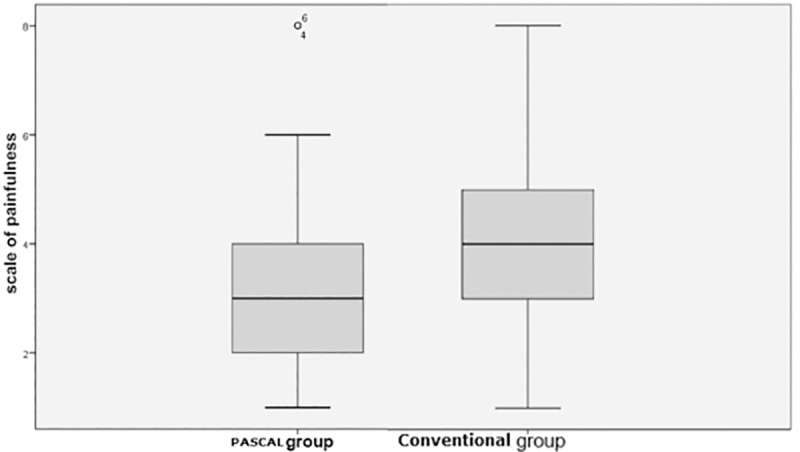
Subjective evaluation of pain intensity–Pascal and Conventional groups.

In the PASCAL group the average value of perception of painfulness on the visual scale of pain (range 0–8, with 8 being the most painful) was 3.28 (median 3, SD 1.9), and in the Conventional group was 3.93 (median 4, SD 1.88). This is represented graphically in Fig 2. The difference was statistically significant for the benefit of the PASCAL group (p < 0.05).

### Complications

In 2 eyes of one patient vitreomacular traction syndrome developed in the period between months 6 and 12 from the start of follow-up; pars plana vitrectomy was carried out with subsequent stabilization of the condition. No other complications were observed.

## Discussion

From the standpoint of individual characteristics, the selected cohort can be considered representative. The total numbers of patients included in the ETDRS and DRS studies, however, were higher than in our cohort, but these cohorts were considerably heterogeneous, and these studies also showed a number of shortcomings (absence of examinations of comorbidities, telephone checks, etc.) [6–9]. In addition, these cohorts showed considerable differences in the characteristics of individual patients and used shorter follow-up periods (3–9 months) [10–15]. Comparable complex studies into the use of this pattern laser system within precisely-defined cohorts have been reported in a few publications only, largely by Muqit et al. [16–21]. This makes our paper exceptional.

In our cohort we employed a different number of laser spots to perform PRP complexly in a pattern laser treatment and also using classic conventional therapy. This was because the application of a laser spots with a shorter impulse result in a lesser extent of photocoagulated tissue than a similar laser spot produced by a standard-duration impulse. Not only does it affect the size of the initial laser spot, but also the area of atrophy of the collateral tissues spreads some distance from the site of treatment. A similar conclusion was reached also by other authors who demonstrated that treatment with an initial number of spots of about 1500 is insufficient [10, 20, 22]. They reported that in order to perform PRP and achieve the same aim with the use of patterns and an impulse length of 20 ms, it is necessary to apply initially more than 2000 spots, and in the case of high-risk PDR characteristics as many as 5000–7000 spots to achieve complete regression of the disease and treatment of a sufficient area of the retina [20].

In our cohort in one-year evaluation, stabilization of the clinical condition (an objective finding in the fundus) occurred in 66.6% of treated eyes of 77% of the patients at the end of the follow-up period. Chappelow et al. [10] observed in her cohort with a follow-up period of 6 months an absence of regression or a progression of PDR–i.e. a failure of the first treatment–in up to 73% of the eyes treated by Pascal vs. 34% of the eyes treated with the conventional argon laser. However, at the first treatment she used a clearly lower number of laser spots (on average 1438) for therapy, and in addition her cohort consisted only of eyes with the characteristics of high-risk PDR. Muraly et al. [23] observed regression of the disease after 1 month in 90% of the cases treated by the Pascal system and in as many as 98% cases after 6 months, compared with 64% and 98% respectively for the conventional laser. However, in his cohort he used on average of 2795 laser spots in a Pascal laser photocoagulator versus 1414 spots in the conventional laser. In the ETDRS study, in those eyes in which early full PRP was carried out there was progression into high-risk PDR in 5–18% after 3 years and in 10–30% after 7 years of monitoring [7]. We infer from these data that in our cohort the risk of progression or failure of treatment were similar to those in the above-mentioned studies.

One of the important and frequently neglected parameters of treatment is the perception of pain during intervention. The present cohort demonstrated a significant lower perception of pain for the pattern laser therapy in contrast to the classic treatment. This coincides with the findings of other authors, although the absolute values of the difference are lower in our cohort [12, 19]. Certain disproportions in our study can be explained by the fact that the previously-mentioned authors used on average only 1500 laser spots in the initial treatment [19]. These laser spots covered particularly the paracentral areas and the middle periphery of the retina, which are well tolerated by patients. In the so-called supplementary treatment, pain was not evaluated, but as soon as the application of laser spots is moved into the more distant periphery, patients begin to feel pain intensively. This is then multiplied by application in patterns. Where the whole treatment is applied in one episode, application to the distant periphery clearly cannot be avoided. Thus for evaluation it is important to compare the performance of total therapy and not merely individual incomplete episodes. All complications observed in our cohort were symmetrical and the causes seem to be probably more connected with the principal disease.

Although the advantage of PRP carried out with the patterns and the use of short laser impulses has already been frequently mentioned in the literature, nevertheless with the exception of the British Royal College of Ophthalmology these findings have not been implemented into the recommended procedures of professional societies [24–26]. In the past couple of years there have been reports advocating for use of anti-VEGF as first line treatment in severe NPDR or PDR [27, 28]. Nevertheless, the cost of such therapy prevents its widespread use. Due to this fact laser treatment of DR still remains a valid option and single session treatment brings considerable benefits to all subjects involved in the process.

## Conclusion

The performance of panretinal laser photocoagulation with the use of patterns and of impulses shorter by an order of magnitude during one or up to two therapeutic episodes in severe advanced NPDR and in PDR was perceived as significantly less painful than the performance of conventional photocoagulation of the retina. This treatment allows to save considerable funds of all subjects participating in the process of treatment and is comparable with the classic retinal photocoagulation in terms of functional, anatomical and clinical efficiency.

## Supporting information

S1 Table General characteristics of the cohort. (DOCX)

S2 Table Efficiency of treatment at 12 months follow-up. (DOCX)

S1 File CONSORT 2010 checklist. (DOC)

S2 File CONSORT 2010 flow diagram. (DOC)

S3 File Trial study protocol in the original language. (DOC)

S4 File Trial study protocol translation. (DOC)

S5 File Open Science Framework public repository. (DOC)
