# Supplementary material for: Single session of pattern scanning laser versus multiple sessions of conventional laser for panretinal photocoagulation in diabetic retinopathy: Efficacy, safety and painfulness
Source: PLoS One. 2019 Jul 16;14(7):e0219282. doi: 10.1371/journal.pone.0219282 (PMC6634372; doi:10.1371/journal.pone.0219282)
Supplement: S1 Table — (DOCX) [file pone.0219282.s001.docx]

| Parameters | (n = 60) |
| --- | --- |
| Age – median (25, 75 percentile) | 57 (45,65) |
| Males | 30 (50%) |
| Females | 30 (50%) |
| DM type 1 | 16 (26.6%) |
| DM type 2 | 44 (73.3%) |
| NPDR | 13 (21.6%) |
| PDR beginning | 21 (35%) |
| PDR high risk | 26 (43.3%) |
| DME | 60 (100%) |
| Length of duration DM (years) | 16 (8.24) |
| HbA1c – median (25, 75 percentile) | 8.5% (6.9 9.3) |
| Follow-up period – median (range) | 24 M (12, 48) |

**General characteristics of the cohort.**

N – number of eyes; HbA1c - glycated hemoglobin; M – month.
