# Supplementary material for: Single session of pattern scanning laser versus multiple sessions of conventional laser for panretinal photocoagulation in diabetic retinopathy: Efficacy, safety and painfulness
Source: PLoS One. 2019 Jul 16;14(7):e0219282. doi: 10.1371/journal.pone.0219282 (PMC6634372; doi:10.1371/journal.pone.0219282)
Supplement: S2 Table — (DOCX) [file pone.0219282.s002.docx]

|  |  |  | **stabilization** | **progression** | **failure** | **Total** |
| --- | --- | --- | --- | --- | --- | --- |
| **Method** | Pascal | Number  (percent share) | 21  70.0% | 8  26.7% | 1  3.3% | 30  100.0% |
|  | Conventional | Number  (percent share) | 19  63.3% | 10  33.3% | 1  3.3% | 30  100.0% |
| **Total** |  | Number  (percent share) | 40  66.6% | 18  30.0% | 2  3.3% | 60  100.0% |

**Efficiency of treatment at 12 months follow-up.**
