# Supplementary material for: Single session of pattern scanning laser versus multiple sessions of conventional laser for panretinal photocoagulation in diabetic retinopathy: Efficacy, safety and painfulness
Source: PLoS One. 2019 Jul 16;14(7):e0219282. doi: 10.1371/journal.pone.0219282 (PMC6634372; doi:10.1371/journal.pone.0219282)
Supplement: S2 File — (DOC) [file pone.0219282.s004.doc]

**
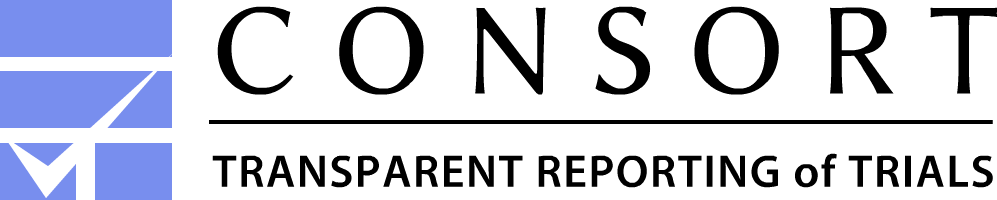
**

**CONSORT 2010 Flow Diagram**

**Allocation**

**Analysis**

**Follow-Up**

**Enrollment**

Assessed for eligibility (n= 30)

Excluded (n= 0 )

♦  Not meeting inclusion criteria (n= 0 )

♦  Declined to participate (n= 0 )

♦  Other reasons (n= 0 )

Analysed (n= 30 )
♦ Excluded from analysis (give reasons) (n= 0)

Lost to follow-up (give reasons) (n= 0 )

Discontinued intervention (give reasons) (n= 0 )

Allocated to intervention (n= 30 )

♦ Received allocated intervention (n= 30 )

♦ Did not receive allocated intervention (give reasons) (n= 0 )

Lost to follow-up (give reasons) (n= 0 )

Discontinued intervention (give reasons) (n= 0 )

Allocated to intervention (n= 30 )

♦ Received allocated intervention (n= 30 )

♦ Did not receive allocated intervention (give reasons) (n= 0 )

Analysed (n= 30 )
♦ Excluded from analysis (give reasons) (n= 0)

Randomized (n= 30 )
