# Supplementary material for: Single session of pattern scanning laser versus multiple sessions of conventional laser for panretinal photocoagulation in diabetic retinopathy: Efficacy, safety and painfulness
Source: PLoS One. 2019 Jul 16;14(7):e0219282. doi: 10.1371/journal.pone.0219282 (PMC6634372; doi:10.1371/journal.pone.0219282)
Supplement: S3 File — (DOC) [file pone.0219282.s005.doc]

# Úvod

Diabetes mellitus je jedním z nejčastějších chronických onemocnění, kterým trpí naše populace. Postiženo je až 7% populace, tj 700 000 nemocných. Diabetická retinopatie je pak nejčastější komplikací diabetu. Po 20 letech trvání diabetu trpí diabetickou retinopatií až 99% diabetiků I. typu a až 60% diabetiků II. typu, V ČR diabetickou retinopatií trpí až 12% diabetické populace. Z toho proliferativní formou trpí každý pátý pacient s diabetickou retinopatií. Prevalence slepoty v souvislosti s diabetickou retinopatií je 3%.

Laserová fotokoagulace sítnice zůstává zlatým standardem léčby posledních desetiletí u diabetické retinopatie, diabetického makulárního edému a mnoha dalších onemocnění sítnice. Panretinální fotokoagulace sítnice se provádí za účelem regrese neovaskularizací a prevenci tvorby nových neovaskularizací. Dosavadní praxe v ČR je užití standardní laserové fotokoagulace sítnice na většině pracovišť, tj ošetření během 3-4 sezení.

PAttern **SCA**n **L**aser je nepřenosný Nd:YAG laser o zdvojené frekvenci 532 nm , který produkuje rychlé krátké série pulsů v přednastavených vzorech jednoduchým sešlápnutím nožního pedálu. Má vestavěný mikroprocesorově řízený scanner, který produkuje širokou paletu vzorů - od jednotlivé stopy až po upravený vzor 56 stop. Časy pulsů jsou 10x – 20x nižší než u standardních systémů, ale celková energie ošetření je ve srovnání se standardními systémy nižší. Stopy jsou pravidelné, více homogenní, dochází k nižšímu kolaterálnímu poškození tkání.

Při ošetření tímto laserem výrazně klesá čas ošetření, subjektivně vnímaná bolest, zvyšuje se komfort ošetření.

Tento laser byl schválen pro použití v USA na konci roku 2005, v Evropě 2006, poprvé byl použit v ČR na Oční klinice FN Ostrava 2007.

V zahraničí pak došlo k určitému rozšíření laseru Pascal, byly také zveřejněny první zkušenosti popisující charakter a zkušenosti s tímto laserem u pacientů. Ve srovnání s předchozí generací laserů tato nová technologie vysoce zvyšuje efektivitu ošetření, preciznost, bezpečnost a komfort pro pacienta i lékaře. Dosud však nebyly publikovány studie v impaktovaných časopisech hodnotící některé níže uvedené parametry za použití statistických metod.

# Cíl projektu

porovnat přínos provádění panretinální laserové fotokoagulace sítnice přístrojem Pascal s využitím všech inovací kompletně během jednoho sezení a provádění panretinální fotokoagulací standardním způsobem během 4 sezení.

Tímto prokázat vyšší funkční aktivitu sítnice po laserovém ošetření Pascalem, nižší subjektivně vnímanou bolestivost ošetření. Dále prokázat bezpečnost z hlediska stability centrální tloušťky sítnice, dále vyšší subjektivně vnímaný status zrakových funkcí po ošetření.

Potvrdit snížení času nutného ke kompletnímu ošetření systémem Pascal.

Potvrdit nižší celkovou energii aplikovanou během ošetření systémem Pascal.

Zároveň předpokládáme průkaz zachování efektivity ošetření ve smyslu stabilizace zrakové ostrosti.

Bude kvantifikován ekonomický přínos snížení počtu ošetření a to z hlediska finanční zátěže jak zdravotnického zařízení, tak pacienta.

Při úspěchu ve výše uvedených parametrech bude možno formulovat nový algoritmus laserového ošetření nejen diabetické retinopatie.

## Hypotéza

Předpokládáme nižší funkční aktivitu diabetické sítnice již před ošetřením, po ošetření systémem Pascal pak nižší pokles funkční aktivity (oproti ošetření standardním systémem), jelikož z dosud publikovaných studií vyplývá, že při použití Pascalu dochází k menšímu kolaterálnímu poškození tkání.

Z dosud publikovaných i vlastních zkušeností se systémem Pascal i standardního ošetření očekáváme čas jednoho ošetření 3 – 10 min u Pascalu (kompletní ošetření panretinální fotokoagulací), 7 – 20 min u standardních systémů (1/3 – ¼ kompletního ošetření panretinální fotokoagulací).

Předpokládáme nižší nebo stejnou bolestivost u systému Pascal pro kratší čas aplikace jednotlivých stop, ale také vyšší počet bodů aplikovaných během jednoho sezení. Stejně hovoří dosavadní zkušenosti.

Ze základní charakteristiky přístroje a původních studií tvůrců laseru očekáváme snížení celkové energie nutné ke kompletnímu ošetření včetně zachování stability centrální tloušťky sítnice po kompletním ošetření provedeném v jednom sezení.

Očekáváme srovnatelnou stabilitu zrakové ostrosti po ošetření i v porovnání s dosavadními studiemi, zejm. DRS studií, ETDRS studií.

Očekáváme finanční úsporu zdravotnického zařízení, pacienta a v neposlední řadě celého zdravotního systému.

Při potvrzení výše uvedených předpokladů bude možno standardizovat nový algoritmus ošetření.

### Metodika

V letech 2009 – 2011 bude průběžně probíhat nábor pacientů, ošetření a sledování jednotlivých parametrů. Jedná se o prospektivní studii.

Do souboru budou zahrnuti pacienti s diagnozou proliferativní diabetická retinopatie, velmi pokročilá neproliferativní diabetická retinopatie, kteří dosud neabsolvovali laserovou fotokoagulaci periferie sítnice. Zároveň pacienti, kteří nemají jiné pokročilé onemocnění, které by mohlo ovlivnit sledované parametry.

Soubor bude mít minimálně 600 očí, což je dostatečný počet k průkazu většiny cílů.

Bude celkem aplikováno 1500 – 3000 bodů, s energií vytitrovanou do zašednutí stopy, použitím kontaktní čočky Mainster PRP 165, velikostí stopy 200 um nastavené na laseru, v topické anestezii, kontaktním mediem bude metylceluloza.

Oči ošetřené Pascalem s využitím všech inovací budou zařazeny do podskupiny A. Oči ošetřené standardním způsobem budou zařazeny do podskupiny B.

Typ ošetření A bude provedeno laserovým systémem Pascal, s časem aplikace 0,02s, s použitím vzorů, ošetření bude provedeno kompletně během jednoho sezení.

Typ ošetření B bude provedeno laserovým systémem Pascal, s časem aplikace 0,2s, body budou aplikovány po jednom, ve 4 sezeních. V 1. sezení budou ošetřeny dolní kvadranty blízká periferie, ve 2. sezení temporální ohraničení makuly a horní kvadranty blízká periferie, ve 3. sezení nasální periferie, ve 4 sezení fill in a daleká periferie.

Pacienti budou rozděleni do 4 skupin. Pacienti s bilaterálním postižením budou randomizovaně rozděleni do skupin 1-3. Skupina č. 1 bude tvořena pacienty, kde bude 1 oko ošetřeno systémem Pascal a 1 oko standardním způsobem. Skupina č. 2 bude tvořena pacienty, kdy budou obě oči ošetřeny Pascalem, skupina č. 3 bude tvořena pacienty, kdy budou obě oči ošetřeny standardním způsobem. Pacienti s jednostranným postižením budou tvořit skupinu č. 4 a budou randomizovaně ošetřeni Pascalem nebo standardním způsobem.

Ze souboru budou vyřazeni pacienti, u nichž nebude možno dokončit léčbu, dále ti, u nichž kteří budou indikováni k jiné, např. chirurgické léčbě během sledovací doby.

Z hodnocení centrální tloušťky sítnice budou vyřazeni pacienti vyžadující paralelní léčbu diabetického makulárního edému.

Funkční aktivitu sítnice budeme sledovat na standardní Ganzfeld elektroretinografii, hodnocena bude amplituda a latence na skotopickém ERG, tyčinková odpověď.

Čas ošetření budeme měřit stopkami.

Celková energie bude odečtena ze shrnutí po ošetření.

Subjektivní bolestivost bude hodnocena na zrakové Stevensově 10-ti stupňové škále bolesti.

Subjektivní zrakové funkce budou hodnoceny modifikovaným dotazníkem NEI-VFQ-25.

Centrální tloušťka sítnice bude hodnocena na OCT, jednak jako absolutní hodnota, jednak jako objem centrální krajiny.

Ekonomická analýza bude zhodnocena jako finanční úspora zdravotnického zařízení při úspoře času, alternativně pak zvýšená výkonnost, dále bude hodnocena finační úspora pacienta (náklady na dopravu do zdravotnického zařízení, doprovod, ušlý zisk)

Zrakovou ostrost budeme hodnotit na logMAR optotypech.

MUDr. J. Němčanský bude koordinovat činnost týmu.

MUDr. J. Němčanský a MUDr. P. Šmehlík budou indikovat pacienty k laserovému ošetření sítnice, vyšetřovat zrakovou ostrost, provádět laserové ošetření, biomikroskopické vyšetření, hodnotit OCT vyšetření, fotografie fundu, subjektivní dotazníky škály bolesti a NEI-VFQ-25, budou prezentovat výsledky na odborných fórech.

MUDr. J. Staněk bude hodnotit ERG vyšetření, bude prezentovat výsledky na odborných fórech..

MUDr. P. Mašek, CSc. provede ekonomickou analýzu.

K hodnocení budou použity standardní statistické metody, včetně parametrických testů.

## Způsob získávání dat

Čas ošetření bude měřen od nasazení kontaktní čočky do skončení fotokoagulace sítnice. Z celkové úspory času bude vykalkulována úspora nákladů, alternativně navýšení počtu výkonů zdravotnického zařízení.

U všech pacientů budou provedena a zhodnocena následující vyšetření před ošetřením, 3 měsíce po ošetření a 1 rok po ošetření.

1, vyšetření nekorigované zrakové ostrosti, korigované zrakové ostrosti na logMAR optotypech

2, barevná fotografie fundu, biomikroskopické vyšetření fundu,

3, centrální tloušťka sítnice a střední objem centrální krajiny na OCT vyšetření, statisticky bude vyhodnocena změna těchto parametrů po ošetření

4, bude vyplněn NEI-VFQ-25 dotazník a statisticky zhodnocena změna výsledného skore po ošetření

5, bude vyšetřena hodnota glykovaného Hb.

U všech pacientů bude provedeno vyšetření ERG před ošetřením a 4 měsíce po ošetření. Statisticky bude vyhodnocena změna amplitudy a latence tyčinkové odpovědi při skotopickém ERG vyšetření.

Po ošetření bude provedena fotografie shrnutí údajů provedeného ošetření (průměrná energie aplikována během sezení, rozmezí energie, počet bodů, použité vzory).

Po ošetření pacient vyplní dotazník subjektivně vnímané bolesti a skore bude statisticky vyhodnoceno.

Po ošetření pacient vyplní dotazník na ekonomickou zátěž absolvování ošetření.

U všech pacientů bude po ošetření vyplněn a zhodnocen dotazník subjektivního vnímání bolesti.

Všichni pacienti podepíšou informovaný souhlas s laserovým ošetřením sítnice.

### Diskuze

Laserová fotokoagulace sítnice je standardní metodou, jejíž validita byla prokázána několika studiemi a to zejména Diabetic Retinopathy Study (DRS), Early Treatment Diabetic Retinopathy Study (ETDRS), tyto probíhaly v 70. a 80. letech minulého století. Interpretací těchto studií jsou klinická doporučení, které tvoří základ indikačního schematu panretinální fotokoagulace sítnice dodnes. Náš projekt je v souladu s indikacemi panretinální fotokoagulace sítnice, u velmi těžké neproliferativní diabetické retinopatie je tato relativní, opodstatněná v určitých případech a uvedená v doporučených postupech národních odborných společností (např. American Academy of Ophthalmology, britskými vládními doporučeními, atd.). Do nástupu laseru Pascal se příliš mnoho nezměnilo v doporučeních ani inovacích dostupných systémů. Dosud byly publikovány studie o experimentálních účincích laseru Pascal s využitím laboratorních zvířat. V současné době v zahraničí probíhají a byly publikovány první pilotní studie a retrospektivní studie/zkušenosti, které zkoumající bolestivost, časový faktor ošetření, ekonomické dopady u systému Pascal s využitím inovací i jiných systémů s krátkým časem impulzu (až 0,0001s) u pacientů. Tyto mají soubory o desítkách pacientů. Podobná práce byla prezentována řešitelem na odborném kongresu Vejdovského olomoucký vědecký den 3/2008 a nyní je připravována ve formě publikace do recenzovaného časopisu. Další 2 práce týmu s touto tematikou byly přijaty do odborného programu XVI. Výročního sjezdu České oftalmologické společnosti.

Dosud však nebyla publikována studie v šíři navrhované v projektu ani nebyly publikovány a ověřeny některé parametry navrhované v projektu – zejména vyšetření ERG, subjektivní změna vnímání zrakových funkcí měřena dotazníky a kvalitní ekonomická analýza. V tomto spatřujeme největší sílu projektu.

### Další informace

Uvedené parametry jsou schopni sledovat i zpracovat výše uvedení řešitel i spoluřešitelé. Mají zkušenosti s vědeckou a publikační aktivitou, většina má dlouholetou praxi v oboru, praxi práce na klinice i zkušenosti s danou problematikou. Až na řešitele jsou vedoucími pracovníky na klinikách. Osobou řešitele je zapojen i mladý vědec.

MUDr. Němčanský a MUDr. Šmehlík mají několikaleté zkušenosti práce v diabetologické oční poradně a s prováděním diagnostické i léčebné péče (včetně laserové fotokoagulace). MUDr. Staněk má mnohaleté zkušenosti práce v elektrofyziologické laboratoři.

MUDr. Mašek, CSc. po celou svou kariéru publikuje a přednáší na národních i mezinárodních kongresech, podílí se na výchově a vzdělávání mladých lékařů, je místopředsedou odborné společnosti pro kataraktovou a refrakční chirurgii, má dlouholeté zkušenosti s ekonomikou provozu zdravotnického zařízení státního i soukromého typu, je vedoucím pracovníkem (primářem/přednostou/manažerem) přes 20 let.

Na Oční klinice FN Ostrava funguje 3 dny v týdnu diabetologická oční poradna, ve FN Ostrava je také diabetologické centrum, což by mělo zajistit dostatečný počet pacientů do souboru. Zároveň jsme vitreoretinálním centrem, které je schopné řešit případné komplikace.

Současné vybavení – Fundus kamera Zeiss Visupac 450 plus, OCT II Zeiss, ERG, LCD optotypy s mody Snellen, logMAR a dalšími, Pascal Photocoagulator.

### Závěr

Dosavadní zkušenosti řešitele a spoluřešitelů s laserovým ošetřením Pascalem s využitím progresivních inovací ukazují signifikantně nižší bolestivost ošetření, vyšší vnímání komfortu u pacientů a zvýšení počtu aplikovaných bodů během jednotlivých sezení, což bylo prezentováno na odborném kongresu řešitelem projektu.

Očekávaným výsledkem projektu je úspěch ve všech sledovaných parametrech.

U laserového ošetření systémem Pascal tedy předpokládáme nižší bolestivost vyšetření, lepší skore dosažené v dotazníku zrakových funkcí, nižší pokles funkční aktivity na ERG, stabilitu centrální tloušťky sítnice a zejména stabilizaci zrakové ostrosti. Při úspěchu ve všech těchto parametrech bude ověříme validitu nového léčebného algoritmu u diabetické retinopatie a umožníme jeho další rozšíření.

Socioekonomické dopady jsou následující. Významným způsobem se sníží počet návštěv u ošetřujícího lékaře, zvýší se tolerance ošetření, sníží se náklady zdravotnického zařízení, čekací doby, bude možno ošetřit i pacienty, kteří se ze sociálních důvodů nemohou dostavit k opakovaným ošetřením. Zvýší se spádová oblast poskytované péče. V neposlední řadě zůstane zachována efektivita a bezpečnost ošetření.

Předpokládaný okruh příjemců v ČR je až 15 000 pacientů s diabetickou retinopatií (zdroj UZIS, 2004), ve spádové oblasti pracoviště pak 1500 pacientů s diabetickou retinopatií a další stovky pacientů s jinými onemocněními sítnice.

Předpokládáme několik publikací v recenzovaných časopisech, alespoň 1 publikaci v časopise s impakt faktorem. Předpokládáme ověření validity tohoto nového algoritmu ošetření a jeho následné široké rozšíření.

Zároveň budeme moci tento nový algoritmus aplikovat při jiných onemocněních sítnice – např. okluzích sítnicových žil, neovaskulárním glaukomu, atd.

Projekt přispěje k řešení cílů a priorit stanovených IGA a to zejm. ověření validity nových léčebných algoritmů a specifikovat nejefektivnější léčebné postupy u chronických onemocněních a to inovací doporučených léčebných postupů.
