# Supplementary material for: Single session of pattern scanning laser versus multiple sessions of conventional laser for panretinal photocoagulation in diabetic retinopathy: Efficacy, safety and painfulness
Source: PLoS One. 2019 Jul 16;14(7):e0219282. doi: 10.1371/journal.pone.0219282 (PMC6634372; doi:10.1371/journal.pone.0219282)
Supplement: S4 File — (DOC) [file pone.0219282.s006.doc]

**Introduction**

Diabetes mellitus is one of the most common chronic diseases our populations suffer. Up to 7% of the population is affected, ie 700,000 patients. Diabetic retinopathy is the most common complication of diabetes. After 20 years of diabetes, up to 99% of type I diabetics and up to 60% of diabetics II suffer from diabetic retinopathy. In the Czech Republic diabetic retinopathy suffers from up to 12% of the diabetic population. Each fifth patient with diabetic retinopathy suffers from this proliferative form. The prevalence of blindness associated with diabetic retinopathy is 3%.
Laser retinal photocoagulation remains a gold standard for recent decade treatment in diabetic retinopathy, diabetic macular edema and many other retinal disorders. Pancretinal photocoagulation of the retina is performed to regress neovascularization and prevent the formation of new neovascularizations. Current practice in the Czech Republic is the use of standard laser photocoagulation of the retina in most workplaces, ie treatment during 3-4 sessions.
PAttern SCAn Laser is a non-portable Nd: YAG double 532 nm laser that produces fast short pulse series in preset patterns by simply pressing the foot pedal. It has a built-in microprocessor-controlled scanner that produces a wide variety of patterns - from a single track to a modified pattern of 56 feet. Pulse times are 10x - 20 times lower than for standard systems, but overall energy treatment is lower than standard systems. Traces are regular, more homogeneous, there is less collateral damage to the tissues.
When treatment with this laser significantly decreases the time of treatment, the subjectively perceived pain, the comfort of treatment increases.
This laser was approved for use in the US at the end of 2005, in Europe 2006, first used in the Czech Republic at the FN Ostrava 2007 Eye Clinic.
There was a certain extension of the Pascal laser abroad, and the first experience describing the nature and experience of this laser in patients was also published. Compared to the previous generation of lasers, this new technology greatly increases the efficiency of the treatment, precision, safety and comfort for both the patient and the physician. However, studies in impacted journals evaluating some of the following parameters using statistical methods have not yet been published.

**Project goal**

to compare the benefits of pancreatin laser photocoagulation of the retina with Pascal using all the innovations completely during one session, and performing pancreatin photocoagulation in a standard manner during 4 sessions.
To demonstrate higher functional activity of the retina after Pascal's laser treatment, a lower subjective perceived painful treatment. Further demonstrate safety from the point of view of the stability of the central retinal thickness, further higher subjective perceived visual function status after treatment.
Confirm the reduction of the time required for complete Pascal treatment.
Confirm lower total energy applied during Pascal treatment.
At the same time, we expect proof of maintaining the effectiveness of the treatment in terms of stabilizing visual acuity.
The economic benefit of reducing the number of treatments will be quantified, both in terms of the financial burden on both the healthcare facility and the patient.
Upon success in the above parameters, it will be possible to formulate a new algorithm for laser treatment not only of diabetic retinopathy.

**Hypothesis**We assume a lower functional activity of the diabetic retina before treatment, after Pascal treatment, a lower decrease in functional activity (compared to standard system treatment), as published studies suggest that less collateral tissue damage is used when using Pascal.
From the previously published and our own experiences with Pascal and standard treatment, we expect a single treatment of 3 to 10 minutes for Pascal (complete pancreatin photocoagulation treatment), 7 - 20 minutes for standard systems (1/3 - ¼ full pancreatin photocoagulation treatment).
We expect less or equal pain in the Pascal system for shorter track times, but also a higher number of points applied during one session. Similarly, experience has been so far.
From the basic characteristics of the instrument and the original studies of the laser makers, we expect the reduction of the total energy required for complete treatment, including maintaining the stability of the central thickness of the retina after a complete treatment performed in one session.
We expect comparable visual acuity stability after treatment as compared to previous studies, especially DRS studies, ETDRS studies.
We expect financial savings for the health care facility, the patient and, last but not least, the entire health system.
When confirming the above assumptions, a new treatment algorithm can be standardized.

**Methodology**Between 2009 and 2011, patients will be recruited, treated and monitored. This is a prospective study.
The group will include patients with a diagnosis of proliferative diabetic retinopathy, very advanced non-proliferative diabetic retinopathy, who have not yet achieved laser photocoagulation of the retinal periphery. At the same time, patients who do not have any other advanced disease that could affect the monitored parameters.
The file will have a minimum of 600 eyes, which is enough to prove most goals.
There will be a total of 1,500 to 3,000 points applied to the trace, using a Mainster PRP 165 contact lens, a 200 μm laser beam setting, in topical anesthesia, methylcellulose contact medium.
Pascale-treated eyes with all innovations will be assigned to subgroup A. Standard-treated eyes will be assigned to subgroup B.
The type of treatment A will be done by the Pascal laser system, with a time of application of 0.02 seconds, using patterns, the treatment will be done completely during one session.
The treatment type B will be performed by the Pascal laser system, with a 0.2 s application time, the points will be applied in one session, in 4 sessions. In the 1st session the lower quadrants near the periphery will be treated, in the 2nd session the temporal margin of the macula and the upper quadrants near the periphery, in the 3rd session of the nasal periphery, in the 4 sessions fill in and the far periphery.
Patients will be divided into 4 groups. Patients with bilateral disability will be randomized into groups 1-3. Group 1 will consist of patients where 1 eye will be treated with Pascal and 1 eye in a standard manner. Group # 2 will consist of patients with both eyes treated with Pascal, Group # 3 will be patients with both eyes treated in the standard way. Patients with unilateral handicaps will form Group 4 and receive randomized Pascal or standard treatment.
Patients who will not be able to complete treatment will be excluded from the set, those who will be indicated for other, eg surgical treatment during the follow-up period.
From the evaluation of the central thickness of the retina, patients requiring parallel treatment of diabetic macular edema will be excluded.

We will monitor the functional activity of the retina on standard Ganzfeld electroretinography, amplitude and latency at the scotomy ERG, bar response.
We will measure the treatment time with stopwatches.
The total energy will be deducted from the summary after treatment.
Subjective painfulness will be assessed on Stevens' 10-degree pain range.
Subjective visual functions will be evaluated by the modified NEI-VFQ-25 questionnaire.
The central thickness of the retina will be evaluated in the OCT both as absolute value and as the volume of the central landscape.
The economic analysis will be evaluated as a financial saving of the health care facility while saving time, as well as increasing the efficiency, as well as the financial savings of the patient (transport costs to the medical facility, escort, lost profit)
We will evaluate visual acuity on logMAR optotypes.
MUDr. J. Němčanský will coordinate the team's activities.
MUDr. J. Němčanský and MUDr. P. Šmehlík will indicate patients for laser treatment of the retina, to examine visual acuity, to perform laser treatment, biomicroscopic examination, to evaluate OCT examinations, fundus photographs, subjective pain scale questionnaires and NEI-VFQ-25, will present results in expert forums.
MUDr. J. Staněk will evaluate the ERG examination, present the results in professional forums ..
MUDr. P. Mašek, CSc. performs economic analysis.
Standard statistical methods, including parametric tests, will be used for evaluation.

**How to get data**The treatment time will be measured from the use of the contact lens until the retinal photocoagulation is completed. From the total time savings, the cost savings will be calculated, alternatively the increase in the number of healthcare facilities.
For all patients, the following pre-treatment examinations will be performed and evaluated, 3 months after treatment and 1 year after treatment.
1, examination of uncorrected visual acuity, corrected visual acuity on logMAR optotypes
2, fundu color photographs, fundus biomedical examination,
3, the central thickness of the retina and the mean volume of the central landscape in the OCT examination, a statistically evaluated change of these parameters after treatment
4, the NEI-VFQ-25 questionnaire will be completed and a statistically evaluated change of the final score after treatment
5, the glycated Hb value will be examined.
All patients will be screened for ERG before treatment and 4 months after treatment. Statistically, the amplitude and latency of the bar response will be evaluated in the scotological ERG examination.
After the treatment, a photograph will be taken of a summary of the treatment data (average energy applied during the session, energy range, number of points, patterns used).
After the treatment the patient fills the questionnaire with subjectively perceived pain and the score will be statistically evaluated.
After the treatment, the patient completes the questionnaire on the economic burden of completing the treatment.
For all patients, a subjective pain perception questionnaire will be completed and evaluated after treatment.
All patients sign an informed consent to the laser treatment of the retina.

**Discussion**Laser retinal photocoagulation is a standard method whose validity has been demonstrated by several studies, namely the Diabetic Retinopathy Study (DRS), the Early Treatment Diabetic Retinopathy Study (ETDRS), which took place in the 1970s and 1980s. Interpretation of these studies is the clinical recommendation that forms the basis of the pancreatin photocoagulation schematic of the retina until today. Our project is consistent with indications of panretinal photocoagulation of the retina; this is very relative in some cases and is recommended by the National Academy of Ophthalmology (UK Government Recommendations, etc.) in the case of very severe non-proliferative diabetic retinopathy. Until the advent of the Pascal laser, too much has changed in the recommendations or innovations of available systems. To date, studies have been published on the experimental effects of Pascal laser using laboratory animals. At present, the first pilot studies and retrospective studies / experiences that study pain, treatment time, economic impacts on Pascal, and other short-time pulse systems (up to 0.0001s) in patients are under way and published. These have files about dozens of patients. Similar work was presented by the researcher at the Scientific Congress of Vejdovský Olomouc Scientific Day 3/2008 and is now being prepared in the form of a publication in the reviewed journal. Another 2 work of the team on this topic was accepted into the professional program of the XVI Annual Meeting of the Czech Ophthalmological Society.
So far, however, a study of the width proposed in the project has not been published and some parameters proposed in the project have not been published and verified - in particular ERG examination, subjective change in visual function perception, questionnaires and good economic analysis. Here we see the greatest strength of the project.

**More information**These parameters are able to track and process the above mentioned solver and co-investigators. They have experience with scientific and publishing activity, most have many years of experience in the field, practice in the clinic and experience with the given issue. Only the investigators are clinicians. The researcher is also involved with a young scientist.
MUDr. German and MUDr. Schmeich have several years of experience working in a diabetology eye care center and performing diagnostic and medical care (including laser photocoagulation). MUDr. Staněk has many years of experience working in an electrophysiological laboratory.
MUDr. Mašek, CSc. he has published and lectures at national and international congresses, participates in the education and training of young doctors, is a vice chairman of a professional company for cataract and refractive surgery, has many years of experience in the economy of operation of state and private healthcare facilities, is a senior worker (senior / manager) over 20 years.
At the FN Ostrava Eye Clinic there is a diabetological ophthalmology clinic 3 days a week, and at the FN Ostrava is also a diabetes center, which should ensure a sufficient number of patients in the group. At the same time, we are a vitreoretinal center capable of solving any complications.
Current equipment - Zeus Visupac 450 plus, OCT II Zeiss, ERG, LCD optotypes with Snellen, logMAR and others, Pascal Photocoagulator.

**Můžete zadat text či webovou adresu nebo** [**přeložit dokument**](https://translate.google.cz/?tr=f&hl=cs)**.**

[**Zrušit**](https://translate.google.cz/?tr=t&hl=cs)

**10039 zn. nad maximum 5000:**

**PŘELOŽIT DALŠÍ TEXT**

**Conclusion**The experience of the co-researcher and co-researcher with Pascal laser treatment, using progressive innovations, shows a significantly lower treatment pain, a higher perception of comfort in patients and an increase in the number of applied points during each session, which was presented at a congress by the project investigator.
The expected result of the project is success in all monitored parameters.
For Pascal laser treatment, we assume a lower painfulness of the examination, a better score achieved in the visual function questionnaire, a lower decrease in ERG functional activity, stability of the central thickness of the retina and, in particular, the stabilization of visual acuity. When successful in all these parameters, we will validate the validity of the new therapeutic algorithm for diabetic retinopathy and allow it to be further extended.
The socio-economic impacts are as follows. Significantly, the number of visits by the attending physician will be reduced, treatment tolerances will be increased, the cost of the health care facility will be reduced, waiting times will be met, patients who can not be treated for rehabilitation for social reasons. The catchment area of ​​the care provided will increase. Last but not least, the effectiveness and safety of treatment remain.
The estimated number of recipients in the Czech Republic is up to 15,000 patients with diabetic retinopathy (source UZIS, 2004), in the catchment area of ​​the workplace 1500 diabetic retinopathy patients and other hundreds of patients with other retinal diseases.
We assume several publications in reviewed journals, at least 1 publications in an impacted factor journal. We expect validation of the validity of this new treatment algorithm and its subsequent widespread extension.
At the same time, we can apply this new algorithm to other retinal diseases such as retinal vein occlusions, neovascular glaucoma,
The project will contribute to addressing the objectives and priorities set by the IGA, in particular by verifying the validity of the new therapeutic algorithms and specifying the most effective treatments for chronic illnesses, namely the innovations of recommended treatment procedures.
