# Supplementary material for: Single session of pattern scanning laser versus multiple sessions of conventional laser for panretinal photocoagulation in diabetic retinopathy: Efficacy, safety and painfulness
Source: PLoS One. 2019 Jul 16;14(7):e0219282. doi: 10.1371/journal.pone.0219282 (PMC6634372; doi:10.1371/journal.pone.0219282)
Supplement: S5 File — (DOC) [file pone.0219282.s007.doc]

All relevant data for the paper can be found in the Open Science Framework public repository at the following link: <https://osf.io/u87r2/>
